# Supplementary material for: Graphitic Carbon Nitride/MOFs Hybrid Composite as Highly Selective and Sensitive Electrodes for Calcium Ion Detection
Source: Molecules. 2023 Dec 18;28(24):8149. doi: 10.3390/molecules28248149 (PMC10746060; doi:10.3390/molecules28248149)
Supplement: Supplementary file 1 [file molecules-28-08149-s001.zip › molecules-2756106-supplementary.pdf]

Graphitic Carbon nitride/MOFs hybrid composite as highly selective and sensitive electrodes for calcium ions detection

Ahmed S. Abou- Elyazed1,2,†, Shilin Li1,3,†, Gehad G. Mohamed4,5, Xiaolin Li1*, Jing Meng6*, Safa S. EL-Sanafery2*

1 Institute of Intelligent Manufacturing Technology, Shenzhen Polytechnic University, Shenzhen, 518055, P. R. China

2 Chemistry Department, Faculty of Science, Menoufia University, Shebin EL-Kom, 32512, Egypt.

3 Heilongjiang Province Key Laboratory of Laser Spectroscopy Technology and Application, Harbin University of Science and Technology, Harbin 150080, China

4 Chemistry Department, Faculty of Science, Cairo University, Giza, 12613, Egypt.

5 Nanoscience Department, Basic and Applied Sciences Institute, Egypt-Japan University of Science and Technology, New Borg El Arab, Alexandria, 21934, Egypt.

6School of Civil and Environmental Engineering, Harbin Institute of Technology (Shenzhen), Shenzhen, 518055, Guangdong, China

* Correspondence: lixiaolin0427@szpu.edu.cn (X.L.); mengjing@hit.edu.cn (J.M.)

† These authors contributed equally to this work.

Calibration of the new carbon paste electrodes (CPE)

The new modified CPE was calibrated by immersion in a 25 mL beaker containing a 20 mL aliquot of Ca(II) solution with concentrations ranging from 1.0 x 10^{-9} to 1.0 x 10^{-1} mol L^{-1} added while continuously stirring, along with a reference electrode, and the potential was recorded after stabilization to 0.1 mV. In order to create a calibration graph, the recorded potentials were plotted as a function of log [Ca(II)]. The sensors were cleaned and rinsed with distilled water in between measurements to get rid of the memory effect.

Table S1. The effect of electrode content on the performance of modified CPEs.

Ionophore type content (Mg)	Graphite mg	Plasticizer type mL	Concentration range (mol L^{-1})	Slope± SD, mV decade $^{-1}$	R^2 (n = 3)
Ti-MOF<br>0.20	250	TCP<br>0.10	$1.0x10^{-5}$-$1.0x10^{-3}$	23.0 ± 0.55	0.9909
Ti-MOF<br>0.50	250	TCP<br>0.10	$1.0x10^{-4}$-$1.0x10^{-3}$	25.15±0.47	0.999
Ti-MOF<br>1.00	250	TCP<br>0.10	$1.0x10^{-4}$-$1.0x10^{-3}$	22.50 ± 0.76	0.9998
Ti-MOF<br>1.50	250	TCP<br>0.10	$1.0x10^{-6}$-$1.0x10^{-3}$	21.90 ± 0.99	0.993
Ti-MOF<br>2.00	250	TCP<br>0.10	$1.0x10^{-4}$-$1.0x10^{-3}$	21.34 ± 0.63	0.994
Ti-MOF<br>1.00<br>(Electrode I)	250	*o*-NPOE<br>0.10	$1.0x10^{-6}$-$1.0x10^{-3}$	28.15 ±0.47	0.990
Ti-MOF<br>1.00	250	DOP<br>0.10	$1.0x10^{-6}$-$1.0x10^{-2}$	18.5±0.81	0.999
Ti-MOF<br>1.00	250	DBP<br>0.10	$1.0x10^{-6}$-$1.0x10^{-2}$	22.1±0.57	0.980
g-C3N4@Ti-MOF<br>0.2	250	*o*-NPOE<br>0.10	$1.0x10^{-7}$-$1.0x10^{-2}$	25.40±0.43	0.980
g-C3N4@Ti-MOF<br>0.5<br>(Electrode II)	250	*o*-NPOE<br>0.10	$1.0x10^{-7}$-$1.0x10^{-2}$	29.15±0.47	0.999
g-C3N4@Ti-MOF<br>1.00	250	*o*-NPOE<br>0.10	$1.0x10^{-7}$-$1.0x10^{-2}$	23.33±0.43	0.980
g-C3N4@Ti-MOF<br>1.50	250	*o*-NPOE<br>0.10	$1.0x10^{-7}$-$1.0x10^{-2}$	23.0±0.77	0.991

Table S2. Evaluation of the modified electrodes' intra- and inter-day precision and accuracy in pure Ca^{2+} solution, powder Milk, and CAL-MAG drug samples.

Sample	Electrode type	Intra-day				Inter-day		
		Taken µM	Found µM	Recovery (%)	RSD	Found µM	Recovery (%)	RSD
Powder Milk	I	18.15	18.09	99.67	0.78	18.06	99.50	0.98
	II	18.15	18.12	99.83	0.64	18.04	99.39	0.55
		1.815	1.813	99.90	0.34	1.810	99.72	0.78
CAL-MAG	I	666.7	656.0	98.35	0.92	659.0	98.80	0.55
		66.67	66.67	100.0	088	66.61	99.10	0.43
	II	666.7	666.3	99.40	0.85	666.5	99.70	0.65
		66.67	66.61	99.10	0.41	66.66	99.85	0.76
Pure solution	I	1000	979.0	99.97	0.97	999.0	99.90	0.48
		1	0.988	98.80	0.43	0.994	99.40	0.92
	II	1000	986.0	98.60	0.43	986.1	98.60	1.00
		1	0.983	98.30	0.55	0.997	99.70	0.75
